# Subclinical Cardiac Changes Following CPX‐351 Induction in Adults with Favorable/Intermediate‐Risk AML

**DOI:** 10.1002/jha2.70373

**Published:** 2026-08-05

**Authors:** Chezi Ganzel, Avraham Frisch, Ofir Wolach, Yakir Moshe, Baher Krayem, Neta Dor, Jacob M Rowe, Yishai Ofran

**Affiliations:** ^1^ The Hematology and Bone Marrow Transplantation Department The Eisenberg Research and Development Authority Shaare Zedek Medical Center The Hebrew University of Jerusalem Jerusalem Israel; ^2^ Department of Hematology and Bone Marrow Transplantation Rambam Health Care Campus Haifa Israel; ^3^ Institute of Hematology Davidoff Cancer Center Rabin Medical Center–Beilinson Hospital Sackler Faculty of Medicine Tel Aviv University Tel Aviv Israel; ^4^ Bone Marrow Transplantation Unit Department of Hematology Tel Aviv Sourasky Medical Center Tel Aviv Israel; ^5^ Department of Hematology Shaare Zedek Medical Center Jerusalem Israel

**Keywords:** AML, anthracyclines, cardiotoxicity, CPX‐351, ejection fraction, global longitudinal strain, Vyxeos

## Abstract

CPX‐351 is associated with reduced non‐hematologic toxicity, but its cardiac safety is not well defined. We prospectively assessed ejection fraction (EF) and global longitudinal strain (GLS) in younger adults with AML treated with a single CPX‐351‐based induction. Median changes were mild and comparable to standard “7 + 3”. However, 27% developed clinically significant cardiac changes. These findings support prospective cardiac monitoring and the need for comparative studies.

Trial Registration: NCT05599360

## 1 Introduction

CPX‐351 (Vyxeos) is a liposomal formulation of daunorubicin and cytarabine approved for newly diagnosed therapy‐related AML and AML with myelodysplasia‐related changes [1]. Liposomal encapsulation prolongs plasma half‐life and enhances bone marrow delivery. This results in reduced non‐hematologic toxicity compared with conventional anthracycline‐based induction. However, given the known cardiotoxicity of anthracyclines [2], the cardiac safety profile of CPX‐351 remains incompletely defined, particularly with long‐term follow‐up.

## 2 Methods

We previously reported a pilot study of CPX‐351 with or without gemtuzumab ozogamicin in 25 adults younger than 60 years with newly diagnosed, FLT3‐ITD‐negative, favorable‐ or intermediate‐risk AML [3]. All patients received a single induction course, with no CPX‐351 consolidation. Echocardiographic assessments, including left ventricular ejection fraction (EF) and global longitudinal strain (GLS), were performed prospectively at baseline, post‐induction, and long‐term follow‐up. Long‐term echocardiographic follow‐up was unavailable in 10 patients due to death (*n* = 5), loss to follow‐up, or patient refusal.

## 3 Results

**FIGURE 1:**
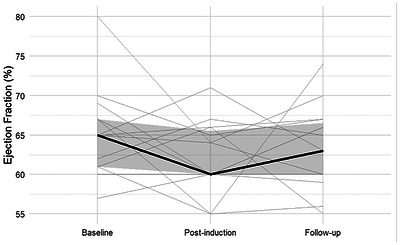
The median EF at different time points.

Fifteen patients had EF measurements at all three time points. The median time from baseline to post‐induction echocardiography was 1.35 months, and from post‐induction to follow‐up was 20.9 months. The median EF at diagnosis, post‐induction, and follow‐up was 65%, 60%, and 63%, respectively (Figure 1).

A multi‐center study of standard “7 + 3” reported a mean EF decline over 2 years from 65.1% to 60.8% (−4.3%) [4], whereas the decline in this cohort was −2%. No patient in this study had an EF < 50%, either post‐induction or at long‐term follow‐up. In contrast, in a cohort reported from the group at Dana‐Farber, 8.5% of 373 patients treated with “7 + 3” had an EF < 50% after induction [5].

**FIGURE 2:**
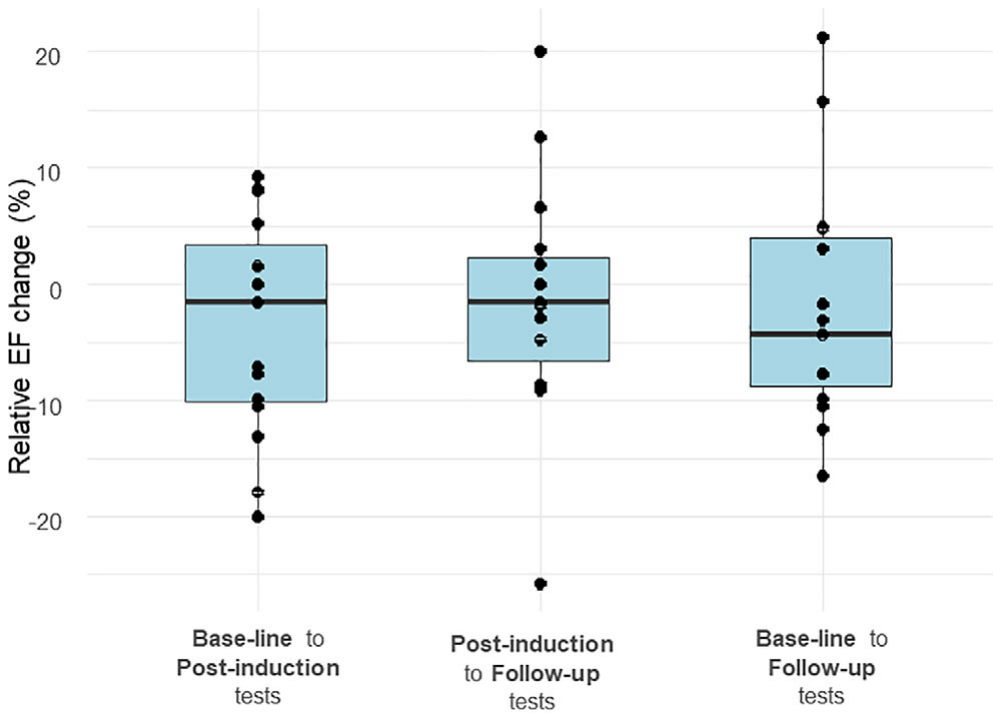
The median relative EF change from baseline.

The median relative EF change was −1.54% from baseline to post‐induction and −4.29% from baseline to follow‐up (Figure 2).

Three patients (20%) experienced a sustained relative EF decline ≥ 10% at follow‐up. Two of these showed marked early EF decline post‐induction (−20.0% and −17.9%) with partial recovery at follow‐up, while the third had a persistent decline (−10.4%)

GLS was available in four patients with complete serial ECHOs. The median relative GLS change from baseline to post‐induction was +19.4%, indicating early subclinical impairment (i.e., GLS becoming less negative). At follow‐up, the median change from baseline was +13.3%. Two patients (50%) showed GLS worsening > 15%, reflected by substantial reductions in myocardial strain post‐induction (+33.6% and +30.8% relative increases), followed by partial improvement at follow‐up (+19.2% and +22.9% relative to baseline). One of these patients also had a relative EF decline ≥ 10%.

## 4 Discussion

Overall, median EF and GLS changes were mild and consistent with subclinical cardiotoxicity. The changes do not appear substantially different from those reported with standard “7 + 3”.

However, four patients (27%) experienced clinically significant cardiac changes, defined by sustained EF decline ≥ 10% and/or GLS worsening > 15%. In these patients, abnormalities were evident shortly after induction. GLS partially recovered at follow‐up, whereas EF remained reduced. This indicates that EF and GLS reflect related but non‐identical manifestations of anthracycline‐related cardiac injury. These findings support continued prospective cardiac monitoring using both EF and GLS in patients treated with CPX‐351. A formal prospective trial is needed, including direct comparison with standard “7+3” induction, to assess the relative cardiotoxicity of CPX‐351. In addition to the small sample size, an important limitation of this study is the relatively young and fit cohort with high baseline EF values and favorable/intermediate‐risk AML. This differs from the broader population typically treated with CPX‐351, particularly patients with adverse‐risk AML. Nevertheless, despite the modest median changes, more than one‐quarter of patients developed clinically meaningful abnormalities that appeared early after induction and persisted partially at long‐term follow‐up, supporting prospective cardiac monitoring for early detection of subclinical cardiac injury.

## Funding

This investigator‐initiated study was supported by Jazz Pharmaceuticals.

## Ethics Statement

Ethics approval was obtained at all participating centers.

## Consent

All patients provided informed consent.

## Conflicts of Interest

The authors declare no conflicts of interest.

## Data Availability

The data that support the findings of this study are available on request from the corresponding author. The data are not publicly available due to privacy or ethical restrictions.
